# Fabrication and Characterization of Electrospun Poly(Caprolactone)/Tannic Acid Scaffold as an Antibacterial Wound Dressing

**DOI:** 10.3390/polym15030593

**Published:** 2023-01-24

**Authors:** Xuefei Chen, Qianqian Zhang, Yi Wang, Jie Meng, Meiqin Wu, Huaizhong Xu, Lei Du, Xiaohua Yang

**Affiliations:** 1 Zhejiang Provincial Research Center of Clothing Engineering Technology, Zhejiang Sci-Tech University, Hangzhou 310018, China; 2 College of Textile Science and Engineering (International Institute of Silk), Zhejiang Sci-Tech University, Hangzhou 310018, China; 3 School of Engineering, Westlake University, Hangzhou 310024, China; 4 Department of Biobased Materials Science, Kyoto Institute of Technology, Kyoto 606–8585, Japan; 5 Zhejiang Carolina Textile Co., Ltd., Quezhou 324299, China

**Keywords:** wound dressing, bio-composite, antibacterial property, nanofibers

## Abstract

Antibacterial wound dressings are promising materials to treat infected skin wounds, which greatly affect the wound-healing process. In this study, tannic acid (TA), a natural antibacterial agent, was successfully loaded by electrospinning into poly(caprolactone) (PCL) fibers in a high concentration. It is suggested that the addition of TA was beneficial for producing uniform and continuous PCL nanofibers. Hydrogen bonds existed between the PCL and TA molecules based on the analysis of FTIR spectra and DSC results. The interactions and continuous network improved the mechanical properties of the scaffolds. Meanwhile, increasing the amount of TA also enhanced the hydrophilicity and water absorption capacity of the scaffold, both of which are beneficial for accelerating wound healing. Moreover, a burst release of the TA in the initial stage and a controlled, steady release behavior over time contributed to the highly antibacterial properties of the PCL/TA scaffolds. The fabrication of the composite scaffold supplies a facile, efficient, and controllable approach to address the issue of antibacterial treatment in wound dressing.

## 1. Introduction

Skin is the largest organ in human body, serving as the first barrier preventing the invasion of various pathogens and external stimuli [1]. Once the skin is damaged by physical, chemical, or heat factors, wound dressings can build an artificial barrier to resist infection. As a crucial part in the field of wound care, the relevant wound dressing products had a global market value of over $20.4 billion in 2021 [2]. Among the various kinds of wound dressings available, antibacterial wound dressings have attracted significant attention, since wounds are often subjected to bacterial infection, especially *Staphylococcus aureus* [3].

Traditional antibiotics are widely used to treat skin infections, although their recurrent use can result in antibiotic resistance [4]. The number of multidrug resistant bacteria has increased at an alarming rate in recent years, accounting for hundreds of thousands of deaths and seriously threatening global public health [5]. As a result, natural antimicrobials extracted from plants have become attractive candidates because of their broad-spectrum antibacterial properties, high security, and efficiency [6]. Tannic acid (TA) is a natural polyphenolic compound extracted from plants, composed of a central glucose connected to five digalloyl ester groups. Various studies have found that TA exhibits antibacterial, antioxidant, anti-inflammatory, and hemostatic properties, which are beneficial for accelerating wound healing [7,8,9]. Additionally, because of its good aqueous solubility, the bioavailability of TA is higher than other natural antimicrobials, such as curcumin, peppermint, cinnamon, and thymol [10,11]. In addition, TA is recognized as a safe additive by the Food and Drug Administration (FDA) [12]. 

As a great potential substitute for common antibiotics, TA-loaded wound dressings in the form of films, hydrogels, and sponges have been designed and investigated. To accelerate wound healing, Taheri et al. used TA as an antibacterial and crosslinking agent to improve the mechanical properties and antibacterial activity of a chitosan/gelatin film [13]. Ninan et al. produced TA-mixed carboxylated agarose hydrogel that displayed favorable antibacterial, anti-inflammatory, and cytocompatible properties [14]. Tan et al. prepared a chitin sponge modified by a complex of TA and calcium ions via layer-by-layer deposition [15]. As a result, the sponge showed antibacterial and hemostatic properties. An ideal wound dressing should be equipped with suitable mechanical properties, a capacity for absorbing extra wound exudates, and good compliance [16]. However, it is difficult for the aforementioned dressings to meet all of these requirements.

In comparison, electrospun nanofibrous scaffolds are a competitive candidate to be used as wound dressings. The basic principle of electrospinning is that a polymer solution is ejected from a capillary tube under an electrical field to a collector to produce a fine fiber network [17]. The high surface-area-to-volume ratio, high porosity, suitable ductility, and tensile strength of electrospun scaffolds can create an ideal microenvironment for cell adhesion, migration, and proliferation [18]. Moreover, the high porosity of electrospun scaffolds can facilitate gas–liquid exchange and avoid excessive drying of the wound, both of which are beneficial for accelerating wound healing. To meet different requirements, the structure and properties of electrospun scaffolds can be tailored by controlling the electrospinning parameters and polymers used.

There have recently been quite a few studies about using electrospun membranes containing TA as antibacterial wound dressings [19,20,21]. To be eligible for use as a dressing for wound healing, the electrospun nanofibrous scaffolds should be biocompatible, and should exhibit good moisture retention and sufficient physical and mechanical strength. Poly(caprolactone) (PCL) is a biodegradable and bioabsorbable synthetic polymer which has been approved by the FDA [22]. Thanks to its favorable biocompatible and mechanical properties, PCL is widely applied in biomedical applications, such as wound dressings, drug delivery systems, and tissue engineering scaffolds [23,24]. However, intrinsically poor hydrophilicity makes it an inappropriate substrate for cell adhesion and proliferation [25]. Modification with a natural hydrophilic material is a great choice for enhancing its cell affinity. In this context, using electrospun PCL nanofibrous scaffolds as a carrier for TA was an attractive choice. 

To our knowledge, information regarding research into the properties of TA-modified PCL nanofibrous scaffolds used as wound dressings is limited. In this context, PCL nanofibrous scaffolds loaded with high concentrations of TA were systematically studied for use as an antibacterial wound dressing. The morphology of the scaffolds with different amounts of TA was analyzed. In particular, the effects of TA on the mechanical, hydrophilic, swelling, and thermal properties of the scaffolds were discussed. Moreover, the release behavior of TA and its antibacterial properties were investigated to further investigate its application as part of an antibacterial wound dressing.

## 2. Experimental Procedures

### 2.1. Materials

Poly(caprolactone) (PCL, Mn = 80,000 g/mol) pellets were purchased from Sigma-Aldrich (Shanghai) Trading Co., Ltd., China. Acetic acid (≥99.8%) and 1,1,3,3,3-Hexafluoroisopropanol (HFIP, 99.5%) were supplied by Aladdin Chemistry Co., Ltd., Shanghai, China. Tannic acid (TA) was obtained from Macklin Biochemical Co., Ltd., Shanghai, China. LB broth powder (FMB grade) and agar (reagent grade) were purchased from Sangon Biotech (Shanghai) Co., Ltd., China. All the materials were used as received.

### 2.2. Preparation of PCL/TA Electrospun Scaffolds

PCL was dissolved in HFIP at a concentration of 10 wt% and TA was dissolved in deionized water at a concentration of 10 wt%. The PCL solution and TA solution were then mixed with varying weight ratios (95:5, 90:10, 80:20). The weight ratios between PCL and TA in the mixed solutions were 9.5:0.5, 9:1, and 8:2, respectively. However, when the weight ratio was 80:20, PCL solution and TA solution could not be mixed homogeneously (Figure S1). In addition, due to the intrinsically low conductivity of PCL, it is difficult to obtain continuous and uniform pure PCL nanofibers by dissolving the PCL in HFIP alone (Figure S2). Therefore, PCL was dissolved in a hybrid solvent of HFIP and deionized water (weight ratio, 75:25) at a concentration of 10 wt% for pure PCL nanofibers. The solutions were stirred for 24 h at room temperature. 

The PCL/TA nanofibrous scaffolds were fabricated by an inhouse-built electrospinning device [26]. The spinning dopes were filled into a 2 mL plastic syringe equipped with a 21-gauge metal needle. High voltages of 10 kV and 12 kV were applied to produce pure PCL and TA-containing PCL nanofibers, respectively. The nozzle-to-collector distance was maintained at 10 cm. The flow rate was controlled at 8 mL/min. PCL/TA nanofiber mats were deposited on a silicon paper resting on an aluminum plate. The spinning lasted 2 h for each sample, at a room temperature of 23 ± 2 °C and relative humidity of 35 ± 2%. The average thicknesses of the mats were 0.098 ± 0.005 mm (PCL), 0.102 ± 0.006 mm (PCL/TA (9.5:0.5)), and 0.101 ± 0.008 mm (PCL/TA (9:1)).

### 2.3. Characterizations

The morphologies of the PCL/TA nanofibers were characterized by field emission scanning electron microscopy (FE-SEM, Ultra 55, ZEISS, Oberkochen, Germany) with an accelerated voltage of 3 kV. The samples were coated with an ultra-thin layer of platinum (JEC-3000 FC, JEOL, Tokyo, Japan) in 100 s at 10 mA before the SEM observation. Fiber diameters were measured by ImageJ (National Institutes of Health, Bethesda, MD, USA) according to the method from Hotaling et al. [27]. More than 100 random fibers from the three captured images were selected in order to analyze their diameters. 

The swelling ratio of the mats was determined gravimetrically by measuring the mass of the sample (2 cm × 2 cm square piece) before and after submerging in deionized water for 24 h at room temperature. The water on the surface of the sample was wiped away using filter paper. The weights of the samples were obtained by electronic balance (FA 2004, Sunny Hengping, Shanghai, China). The measurements were repeated five times using quintuplicate samples. The swelling ratio can be expressed by the following equation:

(1) $$Swelling\;Ratio=\frac{M_{wet}-M_{dry}}{M_{dry}}$$

where *M*_wet_ refers to the mass in the wet state and *M*_dry_ refers to the mass in the dry state.

The water contact angle of the PCL/TA nanofiber mat was monitored via an optical contact-angle instrument (DSA20, KRüSS, Hamburg, Germany) at room temperature. A small amount of deionized water (1 mL) was dropped on the mat’s surface. The contact angle was calculated using images taken at 15 s after the drop was placed. The measurements were repeated three times using triplicate samples.

The mechanical properties of the PCL/TA nanofiber mats were determined by a tensile instrument (KES-G1, KATO, Tokyo, Japan). The samples (5 mm × 20 mm) were cut and then stretched at a tensile rate of 5 mm/min with an initial length of 20 mm at room temperature. The measurements were repeated five times using quintuplicate samples.

The infrared spectra of the PCL/TA nanofiber mats were obtained using Fourier transform infrared spectroscopy (FTIR, NICOLET 5700, NICOLET, Madison, WI, USA). The spectra were recorded from 4000 to 400 cm^−1^ with an average of 32 scans and a resolution of 4 cm^−1^. 

The thermogravimetric analysis of the PCL/TA nanofiber mats was performed using a TA Instruments Discovery TGA (TGA 5500, TA, New Castle, DE, USA). The samples were heated from room temperature to 600 °C with a heating rate of 10 °C/min under a standard air atmosphere.

The melting behaviors of the PCL/TA nanofiber mats were measured by differential scanning calorimetry (DSC) (Q2000, TA, New Castle, DE, USA). All samples (about 3 mg) were sealed in an aluminum pan and were then heated to 200 °C at a scan rate of 10 °C/min in a nitrogen atmosphere.

The actual amounts of TA in the nanofiber mats were determined. The sample (2 cm × 2 cm) was dissolved in a mixture of 85:15 *v*/*v* acetic acid/deionized water, diluted with the same mixture solution. Then, the absorption values at 274 nm were determined using UV–vis spectroscopy (TU-1810PC, PERSEE, Beijing, China) according to the absorption maxima of TA used in the study. The amounts of TA were quantified using the linear equation (y = 24.825x − 0.0613, R² = 0.999, x was the absorption value of UV spectra at 274 nm, y was the concentration of TA in the diluted solution) of a standard curve prepared with TA (5 mg/L, 10mg/L,15mg/L, 20 mg/L, and 25 mg/L). The weights of the samples were obtained by electronic balance (FA 2004, Sunny Hengping, Shanghai, China). The measurements were repeated three times using triplicate samples.

The release of TA from the PCL/TA nanofiber mat was investigated using UV–vis spectroscopy (TU-1810PC, PERSEE, Beijing, China). Each sample was cut into a size of 2 cm × 2 cm and was then submerged in 20 mL deionized water at room temperature. At pre-determined time points ranging from 0 to 72 h, 2 mL of the solution was withdrawn and an equal amount of the fresh deionized water was refilled. The amount of TA released from the PCL/TA nanofiber mat was quantified using UV–vis spectroscopy at 274 nm. The measurements were repeated three times using triplicate samples.

The antibacterial activity of the PCL/TA nanofiber mats against *S. aureus* in vitro was determined by analyzing the zone of inhibition. A fresh preculture of *S. aureus* was adjusted to 1 × 10^6^ CFU/mL and spread uniformly on solid-agar Petri dishes. Then, samples of the PCL/TA nanofiber mats with diameters of 1 cm were put into the Petri dishes and subsequently incubated for 24 h at 37 °C. The inhibition zone was quantified by measuring the diameter. The measurements were repeated three times using triplicate samples.

The results are presented as means ± standard errors. Statistical significance was tested using one-way ANOVA. Statistical significance was accepted at *p* < 0.05 and indicated in the figures as * *p* < 0.05.

## 3. Results and Discussion

### 3.1. Morphology of the PCL/TA Nanofiber Scaffolds

**Figure 1:**
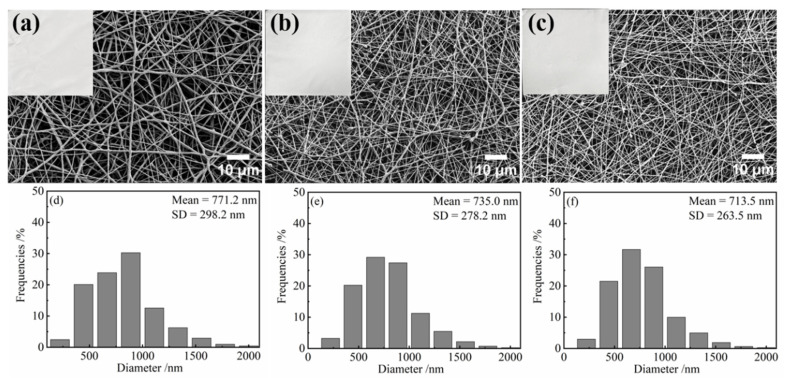
Photos, SEM images, and fiber diameters of electrospun nanofiber scaffolds spun from pure PCL solution (**a**,**d**) and from solutions with PCL/TA weight ratios of 9.5:0.5 (**b**,**e**) and 9:1 (**c**,**f**); the fiber diameter of electrospun PCL/TA (9.5:0.5, 9:1) nanofiber scaffolds are significantly different from the pure PCL nanofiber scaffolds, *p* < 0.05.

The morphology of the PCL/TA nanofiber scaffolds was observed using scanning electron microscopy. As shown in Figure 1, irrespective of the amount of TA, all the scaffolds showed bead-free, randomly oriented, and homogenous morphology. As the concentration of TA increased, the average fiber diameter decreased. Fiber morphology and fiber diameter are strongly influenced by the properties of the spinning solutions used in the electrospinning process, including conductivity, concentration, viscosity, etc. It has been reported that fiber diameter decreases as solution conductivity increases, due to the increased drawing force [18]. Herein, due to the intrinsically low conductivity of PCL [28,29], pure PCL nanofibers showed the largest fiber diameter, at 771.2 ± 298.2 nm. The addition of TA reduced the fiber diameter. The nanofibers produced using PCL/TA (9.5:0.5, 9:1) spinning solution had fiber diameters of 735.0 ± 278.2 nm and 713.5 ± 263.5 nm, respectively. This can be attributed to the higher conductivity of solutions after adding the TA. Simultaneously, the nanofibers became more continuous and uniform as the ratio of TA increased. The slender fibers and continuous network not only strengthen the mechanical properties of the scaffolds, but also benefit the adhesion of cells. 

### 3.2. Chemical Structure of the PCL/TA Nanofiber Scaffolds

**Figure 2:**
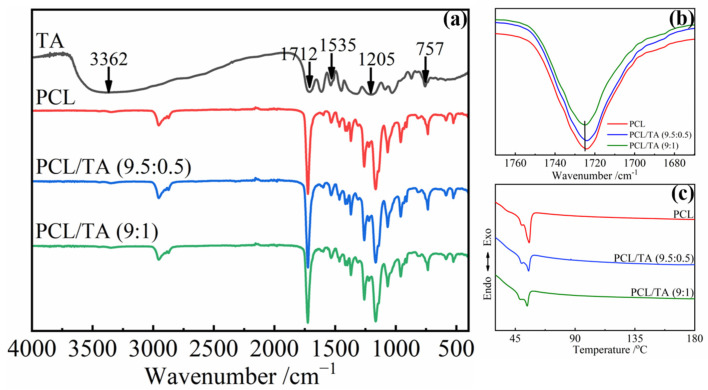
FTIR spectra (**a**,**b**) and DSC curves (**c**) of electrospun nanofiber scaffolds spun from pure PCL and from solutions with different PCL/TA weight ratios (9.5:0.5 and 9:1).

To further understand the chemical structure of the scaffold, the FTIR spectra of the nanofibers were investigated (Figure 2). For the neat TA, a broad absorption band around 3362 cm^−1^ was assigned to the stretching vibration of —OH [30], and the characteristic bands at 1535, 1205, and 757 cm^−1^ corresponded to the stretching of aromatic C=C and C—O bonds, and the bending of aromatic C—H bonds, respectively [13,31]. The bands at 1712 cm^−1^ and 1724 cm^−1^ corresponded to the C=O stretching vibration of TA and PCL, respectively [31,32]. Due to the plentiful catechol and pyrogallol functional groups of TA, it can interact with various species through multiple hydrogen bonds, hydrophobic bonds, π–π bonds, and coordinate bonds [8,9]. In this study, the band from the C=O stretching vibration of PCL exhibited a mild shift from 1724 cm^−1^ to 1725 cm^−1^ after mixing with the TA, especially for the PCL/TA (9:1) nanofibers (Figure 2b). This band shift was attributed to the formation of intermolecular hydrogen bonds between the C=O groups of PCL and the —OH groups of TA [33,34]. The results of DSC on the PCL/TA nanofiber scaffolds also supported this analysis (Figure 2c). The melting point of neat PCL nanofiber scaffolds was at about 55 °C. Because the relatively high solvent evaporation rate during the electrospinning process led to the formation of imperfect spherulitic crystallization, another melting point at around 49 °C was also observed. After adding the TA, the fraction of imperfect crystallization increased as the content of TA increased. This is indicated by the negative effects of TA on the growth of crystallization in the PCL/TA blends. TA can hinder the movement of PCL chains due to the strong interactions between TA and PCL. The results here were in accordance with the previous studies by Yen and Liang, et al. [33,34].

### 3.3. Thermal Properties of the PCL/TA Nanofiber Scaffolds

**Figure 3:**
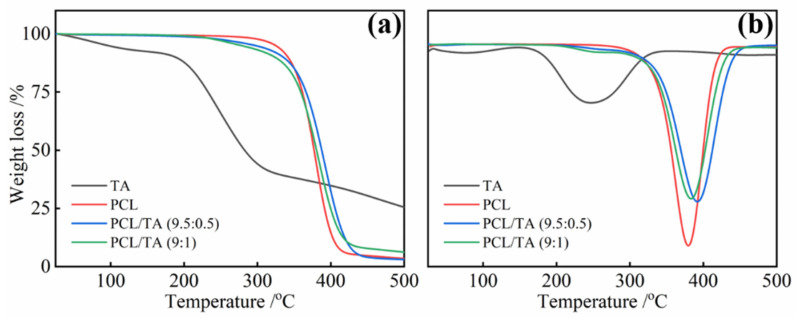
TGA (**a**) and DTG (**b**) of electrospun nanofiber scaffolds spun from PCL from solutions with different PCL/TA weight ratios (9.5:0.5 and 9:1).

Thermogravimetric analysis (TGA) was conducted to confirm the composition of TA in the electrospun nanofibrous mats and to study their thermal stability (Figure 3). In the case of the neat TA, the TGA curve showed multiple degradation processes. The initial decomposition below 150 °C was attributed to the oxidation of TA and water evaporation. Then, a sharp degradation beginning at approximately 150 °C was observed, and the maximum rate of weight loss occurred at about 245 °C (Figure 3b), which is in agreement with values reported in the literature [35,36]. This second zone mainly displayed the depolymerization of TA, which exhibited a mass loss of about 54.2%. Compared with the TA, PCL showed better thermal stability, as the maximum degradation temperature was about 379 °C from the DTG (derivative thermogravimetry) curve. When the TA was mixed with PCL, the initial thermal degradation of PCL/TA nanofiber mats shifted to a lower temperature than pure PCL. Moreover, PCL/TA (9:1) showed higher weight loss during the initial degradation, which confirmed the higher ratio of TA in the bio-composite nanofiber mats. By contrast, when the heating temperature was more than 350 °C, the degradation rate of PCL/TA became slower than that of PCL. This result could be attributed to the formation of intumescent char by TA, which may have acted as a thermal barrier to prevent the pyrolysis of PCL [19,37].

### 3.4. Mechanical Properties of the PCL/TA Nanofiber Scaffolds

**Figure 4:**
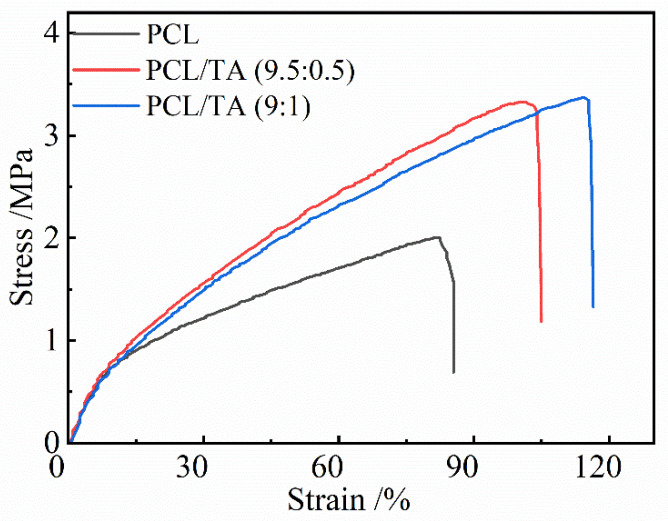
Stress-strain curves of electrospun nanofiber scaffolds spun from PCL and from solutions with different PCL/TA weight ratios (9.5:0.5 and 9:1).

The mechanical properties of electrospun scaffolds are considered to be a substantial factor in wound dressing applications. Various factors, including fibers’ composition, diameter, and morphology, could affect the mechanical performance. Bölgen et al. indicated that thicker PCL fibers can improve the mechanical properties [38]. As shown in Figure 4, PCL nanofiber mats exhibited a lower tensile strength and elongation-to-break than PCL/TA mats, which could be due to it containing noncontinuous nanofibers. The tensile strength and elongation-to-break significantly increased as TA was incorporated into PCL, even though the average diameter decreased; the more continuous 3D network could be responsible for these results. As shown in the Table 1, the tensile strength slightly increased from 3.2 ± 0.4 MPa to 3.5 ± 0.2 MPa when the ratio of TA increased from 5 wt% to 10 wt%. Moreover, the elongation-to-break increased from 105.0 ± 8.2% for PCL/TA (9.5:0.5) to 114 ± 2% for PCL/TA (9:1). Additionally, the intermolecular hydrogen bonds generated between the TA and PCL molecular chains provided crosslinking to enhance the mechanical properties of the mat. Since skin is continually subjected to slight stress, and the tensile strength of skin is reported to be approximately 1.8 MPa [39]. Thus, it is believed that the mechanical properties of the PCL mat loaded with TA is suitable to serve as a wound dressing.

### 3.5. Hydrophilicity and Hygroscopicity of PCL/TA Nanofiber Scaffolds

**Figure 5:**
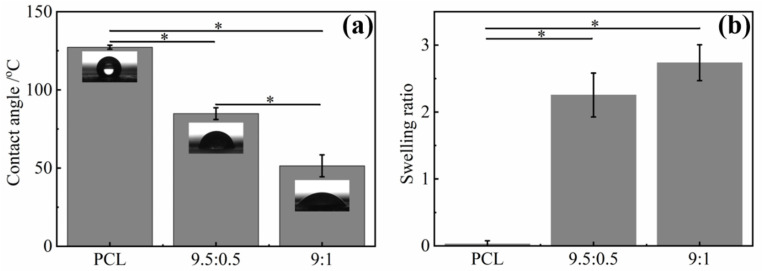
Water contact angle (**a**) and swelling ratio (**b**) of electrospun nanofiber scaffolds spun from PCL and from solutions with different PCL/TA weight ratios (9.5:0.5 and 9:1) (* *p* < 0.05).

The lack of hydrophilicity of PCL is one of the major drawbacks seriously restricting its use as a wound dressing substrate [39,40]. Scaffolds with favorable hydrophilicity and hygroscopicity are beneficial, because they can collect wound exudate and maintain an adequately moist wound environment for cell adhesion and growth [41]. Figure 5a shows that the water contact angle for the PCL nanofiber mats was 127.3 ± 1.3° while the swelling ratio was 0.03. After adding 5 wt% TA, the water contact angle of the mats was 84.8 ± 3.8° (the detailed value is in Table S1). The higher the TA weight ratio was, the smaller contact angel of the PCL/TA nanofiber mat was. Szewczyk et al. reported that the water contact angle of hydrophilic polymers decreased with increasing surface roughness, which was proportional to the fiber diameter [42]. For the hydrophilic PCL/TA nanofibers, the water contact angle should have increased, because the average diameter became smaller as the amount of TA increased, resulting in lower surface roughness. However, the water contact angle of PCL/TA (9:1) nanofiber mats decreased compared to that of the PCL/TA (9.5:0.5) nanofiber mats. The main reason for this could be attributed to the increased amount of TA, which led to the more hydrophilic groups being exposed on the surface of the fibers. The swelling ratio displayed a similar trend, which was enhanced obviously after adding the TA. The hydrophilic nature and interconnected porous structure of the PCL/TA nanofiber scaffold were responsible for the high and fast rate of water uptake [43]. 

### 3.6. TA-Release Behavior of the PCL/TA Nanofiber Scaffolds

**Figure 6:**
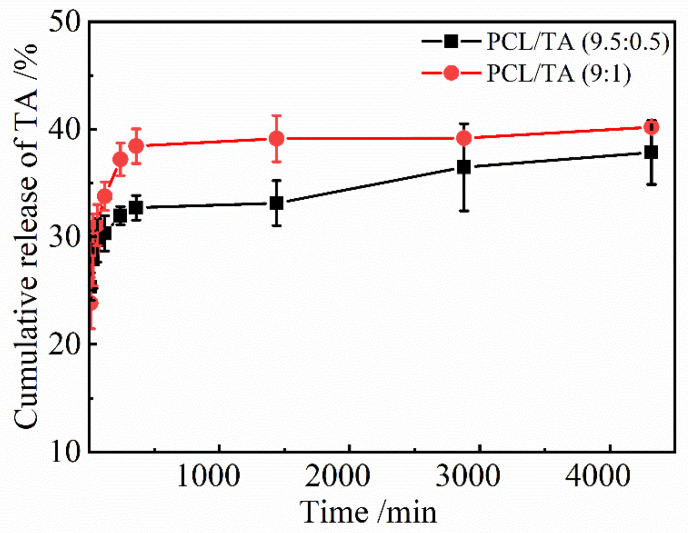
TA release from PCL/TA nanofiber scaffolds spun from solutions with different PCL/TA weight ratios (9.5:0.5 and 9:1) in deionized water.

Because the antibacterial properties of PCL/TA nanofiber scaffolds are the result of the release of TA, it is necessary to study the release behavior of TA in the scaffolds, which has been shown in Figure 6. The actual content of TA in the PCL/TA nanofiber scaffolds was first determined as the base value to calculate the amount of TA released. The actual percentages of TA in the PCL/TA 9.5:0.5 and 9:1 nanofiber scaffolds were 4.70% and 9.48%, respectively, which were close to the mass concentrations of the electrospinning solutions. The cumulative amounts of released TA increased rapidly at first, and then the subsequent rate of release was lower. Radisacljevic et al. showed a similar drug-release behavior of electrospun PCL fibers loaded with cefazolin [44]. In the initial stage, about 29.7 ± 2.0% TA for the PCL/TA (9.5:0.5) scaffold and 31.1 ± 1.9% TA for the PCL/TA (9:1) scaffold were released in the first 60 min. Due to the low molecular weight of TA and its affinity for the aqueous solvent, TA tended to accumulate on the surface of the nanofibers during electrospinning [45]. The initial burst release of TA owed to a rapid diffusion from the surface of the nanofibers. Then, the cumulative amount of released TA increased slowly, tending to a saturation level. This slow release process could be based on drug diffusion and the polymers’ erosion by hydrolysis. On the other hand, the release rate for PCL/TA (9:1) was more rapid than those of PCL/TA (9.5:0.5). Contributing factors may include the smaller fiber diameter and the greater swelling ratio, resulting in a shorter drug-diffusion route and faster hydrolysis rate [46]. These results indicate that the drug release of nanofiber scaffolds can be tailored by the underlying structure and properties.

### 3.7. Antibacterial Properties of PCL/TA Nanofiber Scaffolds

**Figure 7:**
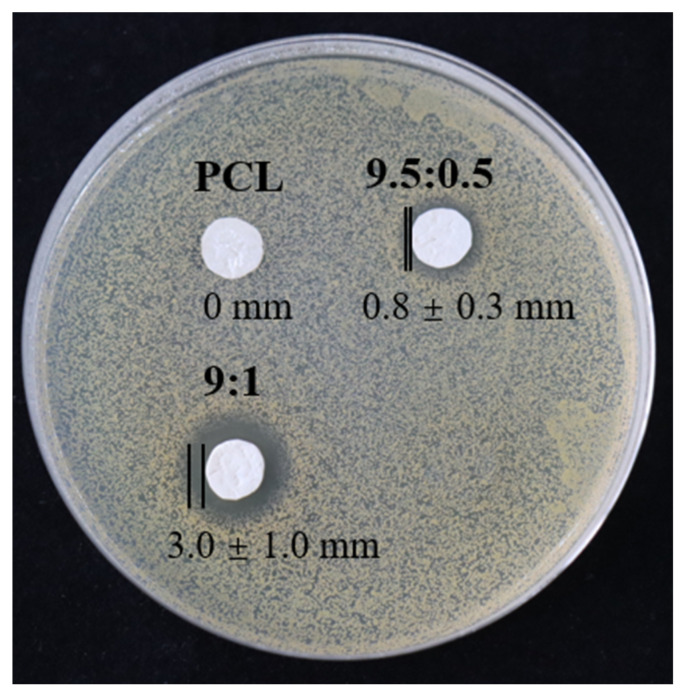
Antibacterial properties of nanofiber scaffolds spun from PCL and from solutions with different PCL/TA weight ratios (9.5:0.5 and 9:1).

To evaluate the antibacterial properties of the PCL/TA nanofiber scaffolds, measurements of the inhibition zone were investigated (Figure 7). As expected, in the case of the pure PCL scaffold, it is obvious that the PCL nanofiber mat did not show any inhibition zone. In contrast, the PCL/TA nanofiber mats exhibited antibacterial properties against *S. aureus*. A narrow inhibitory zone (0.8 ± 0.3 mm) (two other measurements in Figure S3) was observed around the PCL/TA (9.5:0.5) electrospun mat. In comparison, the average inhibition zone for PCL/TA (9:1) can reach up to 3.0 ± 1.0 mm. This observation confirms that the addition of TA improved the antibacterial properties of the nanofiber scaffolds. As observed from the drug-release profile (Figure 6), about 33.1 ± 1.9% of the total amount in the PCL/TA (9:1) nanofiber scaffold was delivered over a 24 h time interval. This release amount seemed to be sufficient to inhibit the bacterial growth.

## 4. Conclusions

In summary, PCL/TA nanofibrous bio-composite scaffolds were successfully prepared as an antibacterial wound dressing. The addition of TA was beneficial to fabricating finer and more continuous nanofibers, by enhancing the conductivity of the spinning solution. According to the analysis of FTIR spectra and DSC curves, there was no chemical reaction, but rather the formation of hydrogen bonds between PCL and TA molecular chains. The continuous nanofibers and the interactions between PCL an TA improved the mechanical properties, contributing to the ability of the scaffolds to sustain external stress. Moreover, the addition of TA enhanced the hydrophilicity and water uptake of the scaffolds, which was favorable in facilitating wound healing. In addition, a rapid release of TA in the first 60 min and a following slow, controlled release were observed. The amount of TA released from the PCL/TA (9:1) nanofiber scaffold over a 24 h time interval seemed to be sufficient to inhibit bacterial growth. Such bio-composite scaffolds with improved properties show great potential for antibacterial wound dressing. For accelerating wound healing, future work will focus on the smart, controlled release of drugs based on the condition of the wound being healed.

## Figures and Tables

**Table 1:** Mechanical properties of electrospun nanofiber scaffolds spun from PCL and from solutions with different PCL/TA weight ratios (9.5:0.5 and 9:1); the tensile strength and elongation-to-break of electrospun PCL/TA (9.5:0.5, 9:1) nanofiber scaffolds are significantly different from those of the PCL nanofiber scaffolds, *p* < 0.05.

Sample	Tensile Strength (MPa)	Elongation-to-Break (%)
PCL	2.0 ± 0.1	88.2 ± 9.4
PCL/TA (9.5:0.5)	3.2 ± 0.4	105 ± 8.2
PCL/TA (9:1)	3.5 ± 0.2	114 ± 2.0

## Data Availability

The data presented in this study are available on request from the corresponding author.

## Supplementary Materials

The following supporting information can be downloaded at: https://www.mdpi.com/article/10.3390/polym15030593/s1.
