# Supplementary material for: Fabrication and Characterization of Electrospun Poly(Caprolactone)/Tannic Acid Scaffold as an Antibacterial Wound Dressing
Source: Polymers (Basel). 2023 Jan 24;15(3):593. doi: 10.3390/polym15030593 (PMC9921954; doi:10.3390/polym15030593)
Supplement: Supplementary file 1 [file polymers-15-00593-s001.zip › polymers-2082301-supplementary.pdf]

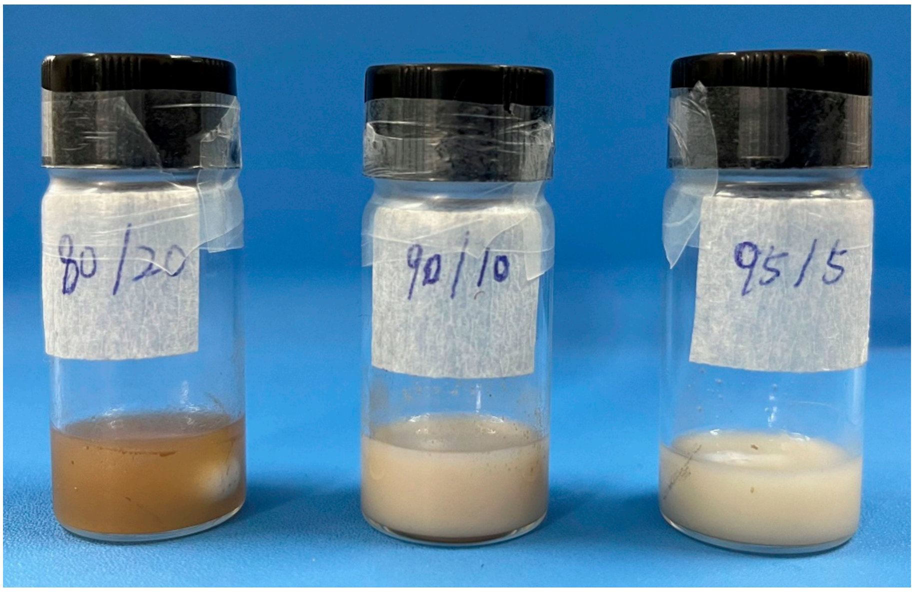

Figure S1 The PCL/TA solution with different weight ratios (9.5:0.5, 9:1 and 8:2).

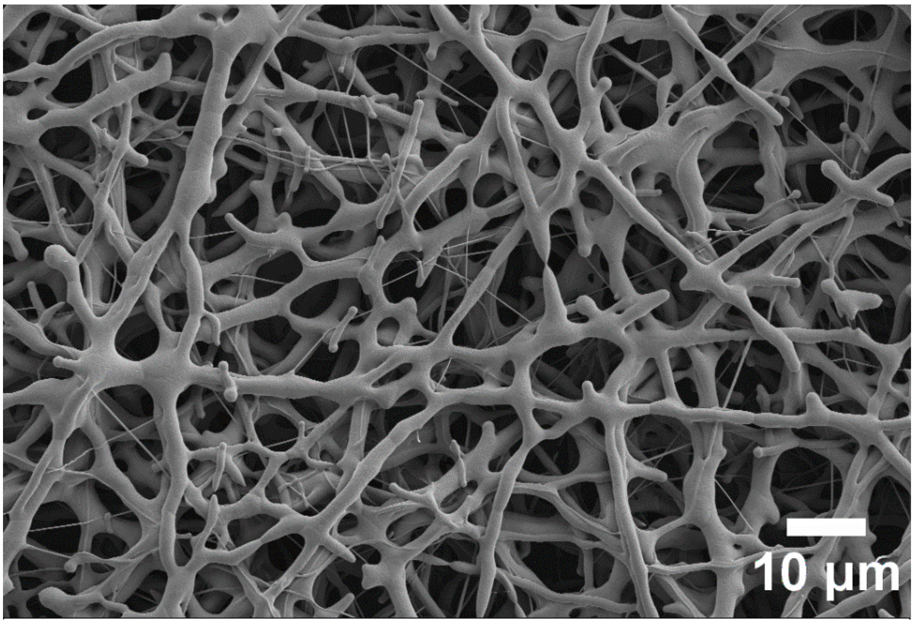

Figure S2 SEM image of electrospun pure PCL using the HFIP alone.

Table S1. Water contact angle and swelling ratio of electrospun PCL and PCL/TA nanofiber scaffolds from solutions with different PCL/TA weight ratios 9.5:5 and 9:1.

Sample	Water contact angle (O)	Swelling ratio
PCL	127.27 ± 1.26	0.03 ± 0.04
PCL/TA (9.5:0.5)	84.83 ± 3.77	2.25 ± 0.32
PCL/TA (9:1)	51.43 ± 7.03	2.73 ± 0.27

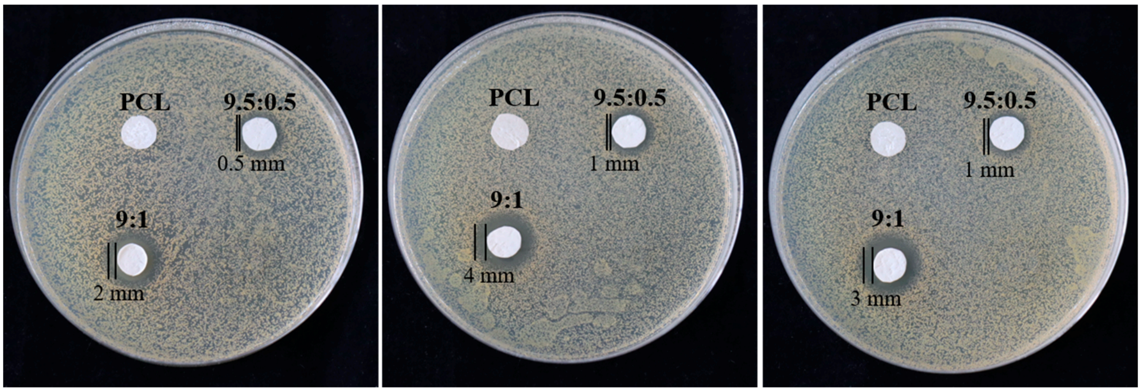

Figure S3 Antibacterial property of PCL and PCL/TA nanofiber scaffolds from solutions with different PCL/TA weight ratios 9.5:0.5 and 9:1.
